# Maternal Calcium Intake at 36 Weeks' Gestation and Pre‐Eclampsia Risk—A Cohort Study

**DOI:** 10.1111/1471-0528.18091

**Published:** 2025-02-11

**Authors:** Anastasija Arechvo, Argyro Syngelaki, Laura A. Magee, Sophie E. Moore, Andrew Doel, Alan Wright, Kypros H. Nicolaides, Peter von Dadelszen

**Affiliations:** ^1^ Harris Birthright Research Centre for Fetal Medicine King's College London UK; ^2^ Department of Women and Children's Health, School of Life Course and Population Sciences King's College London London UK; ^3^ Institute of Health Research University of Exeter Exeter UK

**Keywords:** calcium, deprivation, maternal nutrition, pre‐eclampsia

## Abstract

**Objective:**

The objective of this study is to examine the association between dietary calcium intake (Ca) and pre‐eclampsia (PE).

**Design:**

Cohort study.

**Setting:**

Inner‐city hospital.

**Population:**

A total of 2838 women with singleton pregnancies at 35^+0^–36^+6^ weeks' gestation, including 96 (3.4%) who subsequently developed PE.

**Methods:**

Online 24‐h dietary recall questionnaire was used to measure Ca intake. In the low (< 700 mg/d) vs. adequate (≥ 700 mg/d) Ca intake groups, we compared the prevalence of PE‐associated maternal risk factors and the incidence of PE. In multivariate regression, we examined the low Ca intake and PE relationship, adjusted for established PE risk factors (including blood pressure and angiogenic biomarkers) and any additional factors associated with low Ca intake specifically.

**Main Outcome Measure:**

PE.

**Results:**

Overall, 405 (14.3%) women had low Ca intake. Low (vs. adequate) Ca intake was associated with a higher incidence of PE (6.2% vs. 2.9%; odds ratio 2.2, 95% confidence interval 1.3–3.7), as well as more prevalent risk factors for PE, including Black ethnicity (34.1% vs. 11.8%), South Asian ethnicity (10.1% vs. 7.2%), high body mass index (29.8 vs. 28.3 kg/m^2^) and more deprived index of multiple deprivation (54.3% vs. 35.5%). In multivariate regression adjusting for other PE risk factors, low Ca intake was no longer associated with PE (OR 1.7, 95% CI 0.9–3.2).

**Conclusions:**

Although some contribution from low Ca to the development of PE cannot be ruled out, after accounting for maternal characteristics, medical history and deprivation, low Ca intake did not make an independent contribution to the development of PE in this population of mixed‐ethnicity women.

## 1 Introduction

Pre‐eclampsia (PE), which complicates ~5% of pregnancies worldwide, is responsible for ≈46 000 maternal and > 500 000 perinatal deaths annually [1]. Although the risk of these adverse outcomes is higher for women with preterm (vs. term) PE with delivery at < 37 weeks, term PE is three times more common than preterm PE, making the contribution of term PE to adverse outcomes substantial [2]. Furthermore, first‐trimester multifactor screening is effective in the prediction of preterm PE, but not term PE, and aspirin use by women at high risk of PE prevents 60% of preterm PE, but is stopped at 36 weeks' gestation and does not reduce the incidence of term PE [3]. Consequently, there is a need for an additional strategy to prevent PE, especially term PE.

Epidemiological data suggest that there is an inverse correlation between dietary calcium (Ca) intake and the incidence of the hypertensive disorders of pregnancy, across diverse populations [4, 5]. Furthermore, meta‐analyses of several randomised trials have reported that Ca supplementation reduces the risk of PE by more than 50%, especially in countries with low Ca intake [6, 7]. As such, the World Health Organization recommends 1.5‐2 g elemental Ca daily from 20 weeks' gestation, for pregnant women with low dietary Ca intake [8]. However, **i**n the three largest trials accounting for 88% of total recruitment in the Cochrane and WHO reviews [6, 8], there was no significant reduction in the incidence of PE with Ca supplementation and no evidence of heterogeneity in treatment effect between studies (*I* ^2^ = 0%) [9]. With inclusion of 10 smaller studies, the mean relative risk decreased to 0.45, but between‐trial heterogeneity became substantial (i.e., *I* ^2^ of 70%). The authors concluded that in the assessment of the effect of Ca supplementation on PE risk, a focus on the mean of the random‐effects meta‐analysis is misleading in the presence of such substantial heterogeneity [9].

In this cohort study of women undergoing routine assessment at 35^+0^–36^+6^ weeks' gestation, in an ethnically and socioeconomically diverse population in London, UK, we sought to understand the interrelationships between dietary Ca intake and PE incidence.

## 2 Methods

### 2.1 Study Participants

This was a prospective observational cohort study of women with singleton pregnancies who attended the Harris Birthright Research Centre for Fetal Medicine, King's College Hospital, London UK, for a routine clinical assessment at 35^+0^–36^+6^ weeks' gestation. This visit included recording of maternal demographic characteristics and medical history, ultrasound examination for foetal anatomy and growth, measurement of mean arterial pressure (MAP) by validated automated devices and a standardised protocol [10], and measurement of serum concentration of placental growth factor (PlGF) and soluble fms‐like tyrosine kinase‐1 (sFLT‐1), in pg/mL, by an automated biochemical analyser (BRAHMS KRYPTOR compact PLUS, Thermo Fisher Scientific, Hennigsdorf, Germany).

At this routine visit, women were invited to participate in a study involving dietary intake assessment. Those agreeing to participate provided written informed consent for the study, which was approved by the National Health Service Research Ethics Committee (see “Details of ethics approval”).

We used the index of multiple deprivation (IMD) as a measure of socioeconomic status. IMD is derived from the booking postcode and it is designed to identify aspects of deprivation across seven domains: (i) income, (ii) employment, (iii) education, skills and training, (iv) health and disability, (v) crime, (vi) barriers to housing and services and (vii) living environment [11]. These domains are combined, using appropriate weights, to calculate the IMD for each neighbourhood in England. Each neighbourhood is then ranked according to their level of deprivation relative to that of other areas, with categorisation into one of 10 equal groups, with decile 1 containing those areas that are in the 10% most deprived and decile 10 containing areas that are in the 10% least deprived.

Included in the study were women: with a singleton pregnancy, carrying a live foetus and who were able to provide written informed consent. Excluded were women who were < 16 years of age or unable to consent (e.g., those who were unconscious or very ill, with serious mental illness or not fluent in English in the absence of an interpreter); subsequently, exclusions were extended to women who subsequently gave birth to newborns with aneuploidies or major foetal abnormalities. Lack of internet access was not an exclusion criterion because our researchers conducted telephone interviews with such women. Gestational age was determined by ultrasound using first‐trimester crown‐rump length [12].

### 2.2 Dietary Intake Assessment

Women were asked to complete 24‐h dietary recall, using the Intake24 Online Dietary Recall Tool (https://intake24.co.uk). This is an open‐source, self‐completed, computerised dietary recall system that offers similar data quality to interviewer‐led recalls at a significantly lower cost [13]. It is a practical and cost‐effective approach for assessing habitual dietary intake over long periods. The Intake24 food database includes translation of all labels, prompts and contextual help messages and an adaptable food database. Intake24 provides an online collaborative editing tool for authoring localisations and automated data upload. Intake24 was introduced into the UK National Diet and Nutrition Survey (NDNS) Rolling Programme from October 2019 (https://www.gov.uk/government/collections/national‐diet‐and‐nutrition‐survey).

All women were asked to complete the online Intake24 questionnaire three times, each taking approximately 15 min: (i) at the time of their 35^+0^–36^+6^ week hospital visit’ (ii) two to three days later and (iii) on the first Sunday or Monday after their 35^+0^–36^+6^ week appointment. Previous studies have suggested that one dietary recall is insufficient to evaluated habitual dietary intake and two to four recalls using Intake24 appear to be sufficient for assessment at the group level [14, 15]. Our process is similar to the NDNS, which undertakes multiple Intake24 dietary recalls typically over several days, often including both weekdays and weekends.

Also, intake of nutritional supplements was recorded and their Ca content determined using manufacturers' online product information. Women did not receive personalised feedback on their diets, to observe an unbiased relationship between antenatal diet and PE.

In the calculation of Ca intake, we combined information from diet and supplements. In addition, we estimated the Ca intake from water; women provided information on daily volume intake of water and whether this was tap water or bottled mineral water (still or carbonated), and on the basis of this information, the Ca content was calculated. In the case of tap water, it was assumed that the Ca concentration was 120 mg/L (https://thewaterprofessor.com/blogs/articles/what‐causes‐hard‐water‐in‐london).

### 2.3 Outcome Measure

The primary outcome was delivery with PE, defined according to the American College of Obstetricians and Gynaecologists, as chronic or gestational hypertension (i.e., systolic blood pressure ≥ 140 mmHg or diastolic blood pressure ≥ 90 mmHg, on at least two occasions, four hours apart, and developing after 20 weeks' gestation in a previously normotensive women) AND at least one of the following: proteinuria (≥ 300 mg/24 h, protein‐to‐creatinine ratio ≥ 30 mg/mmol or urinary dipstick testing > 2+), renal insufficiency (serum creatinine > 97 μmol/L in the absence of underlying renal disease), hepatic dysfunction (transaminases more than twice the upper limit of normal [≥ 65 IU/L for our laboratory]), thrombocytopaenia (platelet count < 100 000/μL), neurological complications (e.g., cerebral or visual symptoms) or pulmonary oedema [16].

### 2.4 Data Collection

Maternal demographic characteristics, medical and obstetric history and details of pregnancy outcome were recorded in the Viewpoint clinical database, a password‐secured data management program in Fetal Medicine (GE Healthcare GMBH, Solingen, Germany). Dietary and supplement data from Intake24 were stored in a password‐protected database, designed by the Intake24 team and stored on the Cambridge server; the research team members were given access to the web‐based tool to download dietary intake data at any point. Participants were identified only by their study ID, with the list that linked identifiers and study IDs stored only on NHS computers.

### 2.5 Statistical Analysis

The primary purpose of the analysis is to assess the association between low Ca and incidence of PE. A sample size of ~3000 women was estimated to have at least 75 women with term PE for this purpose [17].

Baseline characteristics (including Ca intake as the median derived from completed questionnaires) and pregnancy outcomes were presented descriptively, for the cohort overall, according to Ca intake that is designated to be low for the UK (< 700 mg/day [18]) or adequate (≥ 700 mg/day) and by the number of recalls. Continuous variables were compared between groups by Student's t‐test and categorical variables by chi‐square test, as appropriate. Logistic regression models were fitted, with delivery with PE as the outcome and low Ca intake (< 700 mg/day) as a predictor. Additional covariates were established risk factors for PE, as defined by the FMF competing risks history model [19] or were associated with Ca intake; these risk factors were added one by one according to their contribution to the FMF competing risks model, cumulatively and the odds ratio (OR) and 95% confidence interval (CI) for PE assessed at each stage. The statistical software package R was used for analyses. The modelling was repeated twice, first, for the full data set and then separately for participants with either one/two or three recalls for Intake24, and therefore, measurements of Ca. No adjustment was made for multiple comparisons, as we aimed to understand any relationship between low Ca intake and PE, rather than guide clinical practice.

## 3 Results

### 3.1 Study Participants

From May 2022 to September 2023, we recruited 2838 participants, including 96 (3.4%) who developed PE and 54 (1.9%) who developed gestational hypertension (GH).

Table 1 presents the characteristics of the study population, the PE and GH groups are each compared with the unaffected group. It demonstrates that there were many differences between PE and GH groups, compared with unaffected pregnancies. In the PE and GH groups, compared with the unaffected group, maternal weight and body mass index (BMI) tended to be higher, and women were more often nulliparous or parous with a previous history of PE. In the PE group (vs. unaffected pregnancies), more women had chronic hypertension, there was more social deprivation (i.e., lower IMD deciles [11]), and daily Ca intake was lower, as assessed by food plus supplements, with or without accounting for Ca in water. Vitamin D intake was similar across groups and related primarily to supplementation.

**TABLE 1:** Baseline characteristics and pregnancy outcomes of the study population.

Characteristic	Unaffected (*n* = 2688)	PE (*n* = 96)	GH (*n* = 54)	*p*
Maternal age (years)	34.0 (30.7, 36.9)	33.7 (30.6, 37.0)	35.5 (32.4, 37.5)	0.147
Maternal weight (kg)	78.0 (70.5, 88.0)	85.1 (76.9, 99.9)	85.8 (80.2,100.1)	< 0.0001
Maternal height (cm)	165 (161, 170)	167 (162, 171)	167 (163, 171)	0.229
Body mass index (kg/m^2^)	28.4 (25.8, 31.9)	30.9 (27.5, 35.5)	31.1 (28.3, 35.8)	< 0.0001
Gestational age (weeks)	35.6 (35.3, 35.9)	35.6 (35.4, 35.9)	35.7 (35.4, 36.0)	0.276
Ethnicity				0.235
White	1900 (70.68)	64 (66.67)	40 (74.07)	
Black	393 (14.62)	23 (23.96)	8 (14.81)	
South Asian	208 (7.74)	6 (6.25)	3 (5.56)	
East Asian	74 (2.75)	2 (2.08)	0 (0.0)	
Mixed	113 (4.20)	1 (1.04)	3 (5.56)	
Medical history				
Chronic hypertension	28 (1.04)	5 (5.21)	0 (0.0)	0.011
Diabetes mellitus Type 1	13 (0.48)	0 (0.0)	0 (0.0)	0.133
Diabetes mellitus Type 2	12 (0.45)	0 (0.0)	0 (0.0)	
SLE/APS	8 (0.30)	0 (0.0)	0 (0.0)	1
Smoking	28 (1.04)	2 (2.08)	0 (0.0)	0.353
Family history of PE	78 (2.90)	5 (5.21)	5 (9.26)	0.017
Method of conception				0.172
Natural	2494 (92.78)	88 (91.67)	46 (85.19)	
In vitro fertilisation	175 (6.51)	8 (8.33)	7 (12.96)	
Ovulation drugs	19 (0.71)	0 (0.0)	1 (1.85)	
Parity				< 0.0001
Parous, no previous PE	1273 (47.36)	24 (25.00)	16 (29.63)	
Parous, previous PE	66 (2.46)	10 (10.42)	4 (7.41)	
Nulliparous	1349 (50.19)	62 (64.58)	34 (62.96)	
Inter‐pregnancy interval (yr)	2.5 (1.5, 4.4)	3.0 (2.2, 5.1)	1.9 (1.2, 5.3)	0.664
Index of multiple deprivation				0.065
1 or 2	347 (12.91)	15 (15.62)	4 (7.41)	
3 or 4	676 (25.15)	30 (31.25)	11 (20.37)	
5 or 6	595 (22.14)	28 (29.17)	17 (31.48)	
7 or 8	602 (22.40)	16 (16.67)	13 (24.07)	
9 or 10	468 (17.41)	7 (7.29)	9 (16.67)	
Calcium intake (mg/day)				0.171
Food + supplements	745 (534, 1015)	680 (433, 997)	781 (507, 987)	0.049
Food + supplements + water	1083 (837, 1403)	953 (666, 1293)	1105 (837, 1352)	0.926
Vitamin D intake (μg/day)	12.0 (10.0, 15.0)	11.0 (11.0, 15.0)	12.0 (4.8, 15.0)	< 0.0001

Abbreviations: APS, antiphospholipid syndrome; Ca, calcium; GH, gestational hypertension; IMD, index of multiple deprivation; PE, pre‐eclampsia; SLE, systemic lupus erythematosus.

Table 2 compares the characteristics and outcomes of women with low (< 700 mg/day) vs. adequate (≥ 700 mg/day) Ca intake, from food and supplements (1267, 44.6% [low intake] vs. 1571, 54.5% [adequate intake]) or food, supplements, and accounting for the Ca content of water (405, 14.3% [low intake] vs. 2433, 85.7% [adequate intake]). In low (vs. adequate) Ca intake groups, calculated in either of the two ways described, there were many differences in maternal and pregnancy characteristics: maternal age was lower, BMI higher, and there were more women of Black and South Asian ethnicity, a longer inter‐pregnancy interval, more social deprivation (IMD 1–4 vs. 5–10) and lower intake of vitamin D. In the low Ca group classified including the Ca content of water, there were fewer nulliparous women. With regards to pregnancy outcomes, there were many differences in outcomes, associated with low (vs. adequate) Ca intake, according to food and supplements with/without accounting for Ca in water: more labour induction, earlier gestational age at birth and lower infant birthweight. In the low (vs. adequate) Ca intake group classified by including the Ca content of water, there was a higher incidence of PE and a slightly lower incidence of GH.

**TABLE 2:** Comparison of baseline characteristics and pregnancy outcomes, according to low and adequate daily Ca intake based on diet and supplements, with and without taking into account water intake.

Characteristic	Food and supplementation			Food, supplementation and water		
	Calcium ≥ 700 (*n* = 1571, 55.4%)	Calcium < 700 (*n* = 1267, 44.6%)	*p*	Calcium ≥ 700 (*n* = 2433, 85.7%)	Calcium < 700 (*n* = 405, 14.3%)	*p*
Maternal age (years)	34.2 (31.2, 37.0)	33.7 (30.1, 36.8)	0.0004	34.1 (31.0, 37.0)	33.2 (29.2, 36.7)	0.001
Maternal weight (kg)	78.0 (70.5, 87.4)	79.0 (70.9, 89.8)	0.022	78.0 (70.5, 87.8)	81.4 (72.0, 95.5)	0.0001
Maternal height (cm)	166 (162, 171)	165 (161, 170)	< 0.0001	166 (162, 170)	165 (160, 169)	0.0003
Body mass index (kg/m^2^)	28.2 (25.6, 31.5)	28.9 (26.0, 33.0)	< 0.0001	28.3 (25.7, 31.7)	29.8 (26.6, 34.9)	< 0.0001
Gestational age (weeks)	35.6 (35.4, 35.9)	35.6 (35.3, 35.9)	0.154	35.6 (35.4, 35.9)	35.6 (35.3, 36.0)	0.149
Ethnicity			< 0.0001			< 0.0001
White	1188 (75.62%)	816 (64.40%)		1803 (74.11%)	201 (49.63%)	
Black	160 (10.18%)	264 (20.84%)		286 (11.76%)	138 (34.07%)	
South Asian	115 (7.32%)	102 (8.05%)		176 (7.23%)	41 (10.12%)	
East Asian	45 (2.86%)	31 (2.45%)		68 (2.79%)	8 (1.98%)	
Mixed	63 (4.01%)	54 (4.26%)		100 (4.11%)	17 (4.20%)	
Medical history						
Chronic hypertension	18 (1.15%)	15 (1.18%)	1	27 (1.11%)	6 (1.48%)	0.692
Diabetes mellitus Type 1	5 (0.32%)	8 (0.63%)	0.341	9 (0.37%)	4 (0.99%)	0.246
Diabetes mellitus Type 2	6 (0.38%)	8 (0.63%)	0.497	10 (0.41%)	4 (0.99%)	0.188
SLE/APS	6 (0.38%)	2 (0.16%)	0.445	8 (0.33%)	0	0.516
Smoker	15 (0.95%)	15 (1.18%)	0.683	26 (1.07%)	4 (0.99%)	1
Family history of PE	42 (2.67%)	46 (3.63%)	0.298	75 (3.08%)	13 (3.21%)	0.987
Method of conception			0.679			0.124
Natural	1449 (92.23%)	1179 (93.05%)		2243 (92.19%)	385 (95.06%)	
In vitro fertilisation	111 (7.07%)	79 (6.24%)		172 (7.07%)	18 (4.44%)	
Ovulation drugs	11 (0.70%)	9 (0.71%)		18 (0.74%)	2 (0.49%)	
Parity			0.077			0.001
Nulliparous	829 (52.77%)	616 (48.62%)		1269 (52.16%)	176 (43.46%)	
Parous, no previous pre‐eclampsia	697 (44.37%)	616 (48.62%)		1102 (45.29%)	211 (52.10%)	
Parous, previous pre‐eclampsia	45 (2.86%)	35 (2.76%)		62 (2.55%)	18 (4.44%)	
Inter‐pregnancy interval (years)	2.3 (1.5, 3.9)	2.8 (1.6, 5.1)	0.002	2.4 (1.5, 4.1)	3.3 (1.7, 6.1)	0.008
Index of multiple deprivation			< 0.0001			< 0.0001
1 or 2	175 (11.14%)	191 (15.07%)		278 (11.43%)	88 (21.73%)	
3 or 4	360 (22.92%)	357 (28.18%)		585 (24.04%)	132 (32.59%)	
5 or 6	373 (23.74%)	267 (21.07%)		567 (23.30%)	73 (18.02%)	
7 or 8	367 (23.36%)	264 (20.84%)		565 (23.22%)	66 (16.30%)	
9 or 10	296 (18.84%)	188 (14.84%)		438 (18.00%)	46 (11.36%)	
Vitamin D intake (mcg/day)	12 (11, 15)	11 (6, 14)	< 0.0001	12 (11, 15)	11 (2, 13)	< 0.0001
Biomarkers of preeclampsia						
Mean arterial pressure (mmHg)	85.3 (80.7, 90.7)	85.7 (80.8, 91.3)	0.210	85.4 (80.7, 90.8)	86.3 (81.4, 91.8)	0.045
Placental growth factor (pg/mL)	344 (180, 641)	341 (190, 631)	0.832	342 (185, 635)	355 (189, 647)	0.836
sFLT‐1 (pg/mL)	1925 (1407, 2822)	2008 (1386, 2987)	0.203	1946 (1407, 2898)	2025 (1338, 3006)	0.711
Pregnancy outcomes						
Stillbirth	2 (0.1)	4 (0.3)	0.227	4 (0.2)	2 (0.5)	0.181
Neonatal death	0	0	—	0	0	—
Gestational age at birth (weeks)	39.7 (39.0–40.6)	39.6 (38.9–40.4)	< 0.0001	39.7 (39.0–40.6)	39.4 (38.6–40.3)	< 0.0001
Birthweight (g)	3415 (3110–3730)	3345 (3055–3640)	< 0.0001	3400 (3100–3715)	3270 (3000–3545)	< 0.0001
Admission to neonatal unit > 24 h	41 (2.6)	28 (2.2)	0.492	59 (2.4)	10 (2.5)	0.957
Labour onset						
Spontaneous	864 (55)	654 (51.6)	0.073	1313 (54)	205 (50.6)	0.211
Induced	390 (24.8)	356 (28.1)	0.049	619 (25.4)	127 (31.4)	0.012
No labour	317 (20.2)	257 (20.3)	0.944	501 (20.6)	73 (18)	0.234
Mode of delivery			0.508			0.462
Vaginal	927 (59)	732 (57.8)		1004 (41.3)	175 (43.2)	
Caesarean	644 (41)	535 (42.2)		1429 (58.7)	230 (56.8)	
Pre‐eclampsia	44 (2.80%)	52 (4.10%)	0.070	71 (2.92%)	25 (6.17%)	0.001
Gestational hypertension	29 (1.85%)	25 (1.97%)	0.874	47 (1.93%)	7 (1.73%)	1.000

Abbreviations: Ca, calcium; sFLT‐1, soluble fms‐like tyrosine kinase‐1.

Intake24 was completed three times by 1660 (58.5%) of women, twice by 644 (22.7%) and once by 534 (18.8%). Table 3 compares the characteristics and outcomes of women with three versus those with one or two recalls. In the group with only one or two recalls, the incidence of women of black ethnicity and those with low calcium intake was increased.

**TABLE 3:** Comparison of women with three versus those with one or two recalls.

Characteristics	Three recalls (*n* = 1660)	One or two recalls (*n* = 1178)	*p*
Maternal age (years)	34.2 (31.1, 37.0)	33.6 (30.0, 36.8)	< 0.0001
Maternal weight (kg)	78.0 (70.9, 87.9)	78.8 (70.2, 89.8)	0.153
Maternal height (cm)	166 (161, 170)	165 (161, 170)	0.347
Body mass index (kg/m^2^)	28.2 (25.8, 31.9)	28.8 (25.9, 32.3)	0.051
Gestational age (weeks)	35.6 (35.3, 35.9)	35.6 (35.4, 35.9)	0.065
Ethnicity			< 0.0001
White	1231 (74.16%)	773 (65.62%)	
Black	189 (11.39%)	235 (19.95%)	
South Asian	129 (7.77%)	88 (7.47%)	
East Asian	47 (2.83%)	29 (2.46%)	
Mixed	64 (3.86%)	53 (4.50%)	
Medical history			
Chronic hypertension	26 (1.57%)	7 (0.59%)	0.028
Diabetes mellitus Type 1	8 (0.48%)	5 (0.42%)	1.000
Diabetes mellitus Type 2	3 (0.18%)	11 (0.93%)	0.011
SLE/APS	5 (0.30%)	3 (0.25%)	1
Family history of PE	50 (3.01%)	38 (3.23%)	0.680
Method of conception			0.478
Natural	1531 (92.23%)	1097 (93.12%)	
In vitro fertilisation	115 (6.93%)	75 (6.37%)	
Ovulation drugs	14 (0.84%)	6 (0.51%)	
Parity			0.0002
Parous, no previous PE	715 (43.07%)	598 (50.76%)	
Parous, previous PE	45 (2.71%)	35 (2.97%)	
Nulliparous	900 (54.22%)	545 (46.26%)	
Interpregnancy interval (years)	2.3 (1.5, 4.2)	2.7 (1.6, 4.8)	0.028
Index of multiple deprivation			0.325
1 or 2	198 (11.93%)	168 (14.26%)	
3 or 4	432 (26.02%)	285 (24.19%)	
5 or 6	367 (22.11%)	273 (23.17%)	
7 or 8	377 (22.71%)	254 (21.56%)	
9 or 10	286 (17.23%)	198 (16.81%)	
Ca intake (mg/day)			
Food + supplements	773 (564, 1026)	697 (481, 991)	< 0.0001
< 700 mg/day	674 (40.60%)	593 (50.34%)	< 0.0001
Food + supplements + water	1114 (884, 1416)	1030 (762, 1364)	< 0.0001
< 700 mg/day	176 (10.60%)	229 (19.44%)	< 0.0001
Vitamin D intake (μg/day)	12.0 (11.0, 15.0)	11.0 (8.0, 14.0)	1
Mean arterial pressure (mmHg)	85.6 (81.0, 90.8)	85.3 (80.3, 91.2)	0.224
Placental growth factor (pg/mL)	345 (190, 616)	331 (178, 678)	0.884
sFLT‐1 (pg/mL)	1973 (1432, 2941)	1938 (1339, 2839)	0.224
Hypertensive disorders of pregnancy			0.889
PE	54 (3.25%)	42 (3.57%)	
GH	31 (1.87%)	23 (1.95%)	

Abbreviations: APS, antiphospholipid syndrome; Ca, calcium; GH, gestational hypertension; IMD, index of multiple deprivation; PE, pre‐eclampsia; sFLT‐1, soluble fms‐like tyrosine kinase; SLE, systemic lupus erythematosus.

**FIGURE 1:**
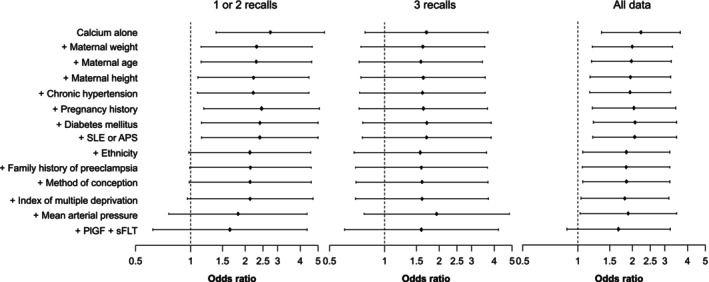
Forest plot of odds ratio with 95% confidence interval for PE of calcium intake < 700 mg/d alone and in combination with maternal characteristics and biomarkers of PE in women who provided one/two recalls (left) or three recalls (middle) or in the whole population (right). Additional covariates were added one by one, cumulatively and the odds ratio (OR) and 95% confidence interval (CI) for PE assessed at each stage. APS, antiphospholipid syndrome; GH, gestational hypertension; IMD, Index of Multiple Deprivation; PE, pre‐eclampsia; PlGF, placental growth factor; sFLT‐1, soluble fms‐like tyrosine kinase; SLE, systemic lupus erythematosus.

Figure 1 provides forest plots of odds ratio with 95% CI for PE of calcium intake < 700 mg/d (including the Ca content of water) alone and in combination with maternal characteristics and biomarkers of PE in the population with one/two recalls using Intake24 (left), those with three recalls (middle) and then the whole population (right). In the whole population in the classification of low Ca intake, which included the Ca content of water, the OR of low Ca intake for PE was 2.2 (95% CI 1.3–3.7). However, addition of other risk factors for PE (i.e., maternal characteristics, MAP and biomarkers) revealed that the OR for low Ca intake reduced to 1.7 (95% CI 0.9–3.2) and no longer made a significant contribution to PE risk. The same pattern was seen in the subgroup of participants with one/two recalls. In the subgroup with three recalls, we observed a similar pattern, in that with the addition of risk factors for PE, the effect of Ca decreased; however, there was no significant contribution to PE risk from low Ca intake, even before adjustment for risk factors for PE (OR of 1.7, 95% CI 0.8–3.7). For details of the individual OR and 95% CI, see Table S1.

## 4 Discussion

### 4.1 Main Findings

In a cohort of ~3000 unselected women from an area of urban London characterised by ethnic diversity and deprivation representative of the UK, we observed that low Ca intake (< 700 mg/day) was common (44%), although less so (14%) when the high calcium content of water in South London was taken into account.

Low Ca intake was associated with many established risk factors for PE, including ethnicity and social deprivation. Although low Ca intake that accounted for the Ca content of water was associated with a doubling in the odds of PE, the association diminished and was no longer significant when other PE risk factors were accounted for in multivariate regression or if Ca intake was assessed among women who provided only one/two Intake24 recalls. Although we cannot rule out some causal effect of low Ca on incidence of PE, we cannot conclude that there is an independent association between low Ca and incidence of PE.

### 4.2 Interpretation of Results and Implications for Clinical Practice

The relationship between dietary Ca intake and PE was first observed in the 1980s in Guatemala [20], where PE incidence is low and dietary intake of Ca high, related to soaking and cooking corn with limewater before grinding [21]. Subsequent observational data have confirmed an inverse relationship between dietary Ca intake and the incidence of PE [4, 5].

Over more than three decades, randomised trials have examined the impact of Ca supplementation on PE incidence. Systematic reviews of these trials have reported that Ca supplementation during pregnancy halves the risk of developing PE [6, 22], but substantial between‐trial heterogeneity in outcomes has remained unexplained despite extensive subgroup analyses, including participants recruited from a low Ca intake population [7]. Importantly, the lack of Ca effect in the three largest trials has led to questions about the effectiveness of Ca supplementation for PE prevention [9].

Our data suggest that the relationship between low Ca intake and PE may be confounded by shared underlying risk factors, such as non‐White ethnicity, social deprivation or high BMI. Alternatively, it is possible that low Ca intake may mediate a relationship between PE risk factors and PE. That said, only three trials enrolled participants from populations of primarily Black women [23, 24, 25], and PE risk was not determined accurately, as with the Fetal Medicine Foundation competing‐risks multivariable models [19]. How low Ca intake and PE risk are related is critically important to understand, to inform discussions about calcium supplementation for PE prevention, an expensive and burdensome intervention in well‐ and under‐resourced settings.

### 4.3 Strengths and Limitations

Strengths of the study include: the prospective examination of a large heterogeneous population of pregnant women, evaluation of dietary Ca intake using a validated tool (Intake24) used by the National Diet and Nutrition Survey, UK; and inclusion in the dietary intake assessment the Ca content of water, which had a major impact on the prevalence of low Ca intake in our population and its association with PE. Importantly, we collected established risk factors for PE, which allowed assessment of the potential independent contribution of low Ca intake to the development of PE.

The main limitation of the study is the necessity to rely on a questionnaire to determine Ca intake, with 59% of women providing one/two recalls. However, serum or plasma levels of calcium do not reflect dietary intake, as levels are homeostatically‐maintained [24]. We assumed that calcium intake was habitual when assessed in the third trimester of pregnancy. We did not assess dietary calcium absorption, which is inversely related to dietary calcium intake and other factors, including those related to PE, such as older age, Black ethnicity or high BMI, although relationships may be complex and affect other aspects of calcium metabolism (such as renal mineral excretion) [26]. We examined Ca intake independent of other micronutrients or dietary pattern. We collected only dietary vitamin D intake and not serum vitamin D which would also reflect vitamin D generation from sun exposure, which would be particularly important in minoritised groups. From a statistical perspective, this is an exploratory analysis and numerous comparisons have been made with no correction for multiplicity, p‐values should be interpreted with this in mind.

### 4.4 Conclusions

Low Ca intake that takes into account the calcium content of water, is associated with an increased risk of PE, as well as other established PE risk factors. When these are taken into account, there is no evidence that low Ca intake makes an independent contribution to development of PE in a population of mixed‐ethnicity women. However, there is uncertainty about the effect of calcium, as reflected in the confidence intervals show in Figure 1. One explanation for why we cannot rule out some effect could be that in our models, we do not fully capture risk factors such as deprivation and that calcium acts as a proxy for this. Whether or not low Ca intake may mediate at least some of the relationship between established risk factors (e.g., deprivation) and PE risk remains to be determined, by work that better defines baseline maternal characteristics, baseline Ca intake and PE risk.

## Author Contributions

A.A., P.v.D. and K.H.N. conceptualised and designed the study. A.A. wrote the first draft of the paper. All authors revised and contributed to the intellectual content of the manuscript.

## Ethics Statement

The study on maternal diet recall received REC approval on the 10th of May 2022 with reference number 22/NW/0126. Measurement of maternal serum concentration of placental growth factor and soluble fms‐like tyrosine kinase‐1 was part of study on prediction of adverse pregnancy outcomes, which received REC approval on the 1st of April 2003 with reference number 02‐03‐033.

## Conflicts of Interest

The authors declare no conflicts of interest.

## Supporting information


Table S1.

## Data Availability

Authors elect to not share data.
