# Supplementary material for: Maternal Calcium Intake at 36 Weeks' Gestation and Pre‐Eclampsia Risk—A Cohort Study
Source: BJOG. 2025 Feb 11;132(6):816–25. doi: 10.1111/1471-0528.18091 (PMC11969911; doi:10.1111/1471-0528.18091)
Supplement: Supplementary file 1 — Table S1. [file BJO-132-816-s001.docx]

**Table S1:** Odds ratio and associated 95% confidence interval for the covariates presented in Figure 1.

|  | **OR (95% CI) for calcium effect on incidence of PE** | | |
| --- | --- | --- | --- |
|  | **All data** | **3 recalls** | **1 or 2 recalls** |
| Calcium alone | 2.22 (1.35, 3.66) | 1.70 (0.78, 3.69) | 2.74 (1.38, 5.43) |
| + Maternal weight | 1.99 (1.20, 3.30) | 1.62 (0.74, 3.54) | 2.30 (1.14, 4.64) |
| + Maternal age | 1.97 (1.19, 3.26) | 1.58 (0.72, 3.45) | 2.29 (1.14, 4.62) |
| + Maternal height | 1.95 (1.17, 3.23) | 1.63 (0.74, 3.58) | 2.21 (1.10, 4.47) |
| + Chronic hypertension | 1.94 (1.16, 3.23) | 1.61 (0.73, 3.57) | 2.21 (1.09, 4.45) |
| + Pregnancy history | 2.03 (1.20, 3.42) | 1.65 (0.74, 3.68) | 2.48 (1.20, 5.11) |
| + Diabetes mellitus | 2.06 (1.22, 3.47) | 1.72 (0.77, 3.84) | 2.47 (1.19, 5.11) |
| + SLE or APS | 2.05 (1.22, 3.46) | 1.72 (0.77, 3.82) | 2.46 (1.19, 5.10) |
| + Ethnicity | 1.85 (1.07, 3.19) | 1.57 (0.69, 3.59) | 2.22 (1.03, 4.74) |
| + Family history of PE | 1.85 (1.07, 3.19) | 1.60 (0.70, 3.66) | 2.23 (1.04, 4.76) |
| + Method of conception | 1.86 (1.08, 3.21) | 1.61 (0.71, 3.68) | 2.22 (1.04, 4.75) |
| + Index of multiple deprivation | 1.81 (1.05, 3.14) | 1.60 (0.70, 3.67) | 2.20 (1.00, 4.83) |
| + Mean arterial pressure | 1.89 (1.03, 3.44) | 1.82 (0.73, 4.54) | 1.88 (0.79, 4.45) |
| + PlGF + sFLT | 1.67 (0.87, 3.19) | 1.48 (0.57, 3.89) | 1.66 (0.63, 4.39) |

APS, antiphospholipid syndrome; CI, confidence interval; OR, odds ratio; PE, pre-eclampsia; PlGF, placental growth factor; sFLT-1, soluble fms-like tyrosine kinase; SLE, systemic lupus erythematosus.
